# Host erythrocyte polymorphisms and exposure to *Plasmodium falciparum *in Papua New Guinea

**DOI:** 10.1186/1475-2875-7-1

**Published:** 2008-01-03

**Authors:** Freya JI Fowkes, Pascal Michon, Lynn Pilling, Ruth M Ripley, Livingstone Tavul, Heather J Imrie, Caira M Woods, Charles S Mgone, Adrian JF Luty, Karen P Day

**Affiliations:** 1 The Peter Medawar Building for Pathogen Research, Department of Zoology, University of Oxford, UK; 2 Department of Medical Parasitology, New York University School of Medicine, New York, USA; 3 Papua New Guinea Institute of Medical Research, Madang, Papua New Guinea; 4 Department of Statistics, University of Oxford, UK; 5 African Malaria Network Trust, Dar es Salaam, Tanzania; 6 Department of Parasitology, Institute for Tropical Medicine, University of Tübingen, Tübingen, Germany; 7 PNG Institute of Medical Research, Madang, Papua New Guinea; 8 Medical Parasitology, MMB-NCMLS, Radboud University Nijmegen Medical Centre, Nijmegen, The Netherlands; 9 European Developing Countries Clinical Trials Partnership, Tygerberg, Cape Town, South Africa

## Abstract

**Background:**

The protection afforded by human erythrocyte polymorphisms against the malaria parasite, *Plasmodium falciparum*, has been proposed to be due to reduced ability of the parasite to invade or develop in erythrocytes. If this were the case, variable levels of parasitaemia and rates of seroconversion to infected-erythrocyte variant surface antigens (VSA) should be seen in different host genotypes.

**Methods:**

To test this hypothesis, *P. falciparum *parasitaemia and anti-VSA antibody levels were measured in a cohort of 555 asymptomatic children from an area of intense malaria transmission in Papua New Guinea. Linear mixed models were used to investigate the effect of α^+^-thalassaemia, complement receptor-1 and south-east Asian ovalocytosis, as well as glucose-6-phosphate dehydrogenase deficiency and ABO blood group on parasitaemia and age-specific seroconversion to VSA.

**Results:**

No host polymorphism showed a significant association with both parasite prevalence/density and age-specific seroconversion to VSA.

**Conclusion:**

Host erythrocyte polymorphisms commonly found in Papua New Guinea do not effect exposure to blood stage *P. falciparum *infection. This contrasts with data for sickle cell trait and highlights that the above-mentioned polymorphisms may confer protection against malaria via distinct mechanisms.

## Background

Human erythrocyte polymorphisms have long been thought to play a role in resistance to malaria. High frequencies of host erythrocyte polymorphisms such as α^+^-thalassaemia, haemoglobin (Hb) S, Hb C, Hb E, complement receptor-1 (CR1) deficiency, glucose-6-phosphate dehydrogenase (G6PD) deficiency and south-east Asian ovalocytosis (SAO) are found in malaria endemic areas. Case-control studies have demonstrated that these polymorphisms reduce the risk of severe falciparum malaria rather than mild malaria [1-22]. Experimentally, research has focused on examining the interactions between the parasite and the erythrocyte to try to understand possible mechanisms of protection of these polymorphisms. *In vitro *experiments have suggested reduced ability of *Plasmodium falciparum *to invade or sustain growth in abnormal erythrocytes [23]. Enhanced clearance of infected erythrocytes by phagocytosis, and increased enhanced susceptibility of *P. falciparum*-infected abnormal erythrocytes to oxidative stress, have also been proposed [24-28]. It has also been hypothesized that α^+^-thalassaemia, CR1 deficiency and blood group O may protect via reduced rosetting [5,21,29-32], the phenomenon whereby non-parasitized erythrocytes bind to parasitized erythrocytes.

It is well documented that individuals with sickle cell trait (HbAS) have lower parasite densities compared to HbAA individuals in asymptomatic, mild and severe malaria [18-20,33-44]. This, together with the observation that the time to reappearance of *P. falciparum *is lower in HbAS individuals after drug treatment [39], provides compelling evidence for a direct interaction of the parasite with the HbS containing erythrocytes or of improved immunity controlling the level of parasitaemia in those with sickle cell trait. However, data on other erythrocyte polymorphisms is less clear cut. Clinical studies have rarely demonstrated differences in parasite density among genotypes in symptomatic malaria. Epidemiological studies in asymptomatic individuals are inconsistent. Cross-sectional studies have shown that α^+^-thalassaemia [45,46], SAO [47,48], G6PD [49-53] and ABO polymorphisms (no studies have examined CR1) have reduced parasite rates/densities while other studies provide no evidence of an effect of these polymorphisms on parasite densities [4,11,54-65]. Data from a single time point may not accurately represent exposure to infection and therefore results of these studies are hard to interpret.

There are two possible ways to measure exposure to malaria blood stage infection: 1) by comparing parasite prevalence/density and, 2) by comparing age-specific antibody seroconversion to major blood stage antigens among host genotypes. Seroconversion to *P. falciparum*-infected erythrocyte variant surface antigens (VSA), which include *P. falciparum *erythrocyte membrane protein-1 (PfEMP-1) and rifins, has been shown to be dependent on the intensity of transmission [66]. Antibody levels in endemic areas reflect the level of IgG seroconversion to *P. falciparum*-infected erythrocyte VSA [67]. Furthermore, a study in Tanzania showed that insecticide-treated bednets limited the repertoire of recognized VSA and reduced antibody levels [68]. Seroconversion can also be considered as a marker for the level of cumulative exposure to the parasite as it increases with age [69]. The more exposure to *P. falciparum *children receive in malaria-endemic areas, the younger children will seroconvert to VSA.

If human erythrocyte polymorphisms were to hamper invasion and/or growth of the parasite or enhance the antibody-dependent control of malarial infection, this should manifest as differences in both mean parasite densities and patterns of age-specific seroconversion to VSA. The frequency of the host polymorphisms α^+^-thalassaemia, CR1, ABO blood group, SAO and G6PD deficiency were determined in a group of children from Papua New Guinea (PNG). Parasitological criteria and levels of anti-VSA antibodies were measured in these children in two consecutive surveys, one year apart. The relationship between these genetic polymorphisms, parasite prevalence/density and age-specific antibody seroconversion to VSA was investigated in a malaria endemic region of PNG.

## Methods

### Study design and data collection

Details of study design and data collection have been reported previously [54]. Briefly, a serial cross-sectional survey was conducted in asymptomatic children living in villages of the Amele region, Madang Province, PNG, where intense year-round malaria transmission occurs [70]. The study took place from November to December of 1999 and 2000 in 566 children aged 1–17 years. A serial study design would ensure that any associations observed with host genotype would be less likely to have occurred by chance. Blood samples were collected using Vacutainer^® ^tubes (Becton Dickinson, UK) and EDTA as anti-coagulant. A small aliquot of whole blood was blotted onto Isocode^® ^Stix (Schleicher & Schuell GmbH, Dassel, Germany) following the manufacturer's instructions, for further human genetic analyses. The remaining blood was centrifuged and separated into plasma (stored at -80°C), buffy coat and erythrocyte pellet, both stored in guanidine hydrochloride at 4°C. 555 paired samples were available for antibody determination.

### DNA polymorphism analysis

α^+^-Thalassaemia genotype was determined by polymerase chain reaction [54]. SAO genotyping was conducted as described earlier [71] using human DNA from Isocode^® ^Stix. G6PD deficiency was evaluated qualitatively (Sigma Diagnostics visual colourimetric assay) and ABO blood groups were determined using anti-A and anti-B antibodies from a kit (BIOSCOT, Edinburgh, Scotland). CR1 genotype was determined by polymerase chain reaction and restriction fragment length polymorphism digest with *Hind*III as previously described with modifications [5,72].

### Parasites

*P. falciparum *isolate PNG/M 12, was collected from a child presenting with acute symptomatic malaria at an aid post in Madang, during a longitudinal cohort study from 1990/91 [73]. Erythrocytes were washed three times after buffy coat depletion in RPMI 1640 media buffered with 25 mM 4-(2-hydroxyethyl)-1-piperazineethanesulfonic acid (HEPES) and supplemented with 25 mM sodium bicarbonate, 2 mM L-glutamine, 300 mM hypoxanthine and 10 μg/ml gentamicin (Gibco, UK) (RPMI-HEPES), and cryopreserved in liquid nitrogen for adaptation in Oxford. Parasites were cultured *in vitro *using the method of Trager and Jensen [74] with modifications [75]. Cells were grown in RPMI/HEPES supplemented with 10% AB serum (from donors residing in a non-malarious area), in an atmosphere of 5% CO_2_, 2% O_2_, and 93% N_2_, sub-cultured into O positive erythrocytes (Oxford Blood Transfusion Service, UK) and stabilates frozen down. Adaptation and maintenance of PfEMP-1 expressing lines were made following a previously published method [67].

### Surface Immunofluorescence Assay (SIFA)

Detection of antibodies bound to the surface of infected erythrocytes (IE) have previously been described [67]. Briefly, trophozoites were synchronized and enriched by Plasmagel^® ^(Laboratoire Roger Bellon, France) flotation[76]. After washing 2× in RPMI and 1× with human tonicity phosphate-buffered saline (HTPBS)/1% BSA (Sigma, UK), trophozoite-IE were adjusted to 10 – 15% parasitaemia. Fifty μl of 1/50 dilution of plasma was added to 5 μl packed cell volume of IE and incubated for 30 mins at RT. All washes between each step were done 3× with HTPBS/1% Bovine serum albumin (BSA). The next antibody layer was a rabbit anti-human IgG (Dako, UK) (1/100 dilution). The final antibody layer was a 1/100 dilution of FITC-coupled swine anti-rabbit IgG (Dako, UK) containing 50 μg/ml ethidium bromide. Cells were fixed for 1 h with 0.5% paraformaldehyde diluted in HTPBS/1% BSA, then washed 1× and resuspended with HTPBS. Samples were read on a FACSCalibur™ flow cytometer (Becton Dickinson, UK): 1000 IE were counted using the program CELLQUEST™ 3.3 (Becton Dickinson, UK) for Macintosh. A hyper immune plasma (HIP) pool from 10 adult residents from Madang and normal human serum (NHS) from three non malaria-exposed Europeans (Oxford Blood Transfusion Service, UK) were used as positive and negative controls, respectively, as previously described [67]. The percentage of IE staining positive for human plasma was calculated relative to the response to HIP and NHS as described earlier [67].

### Statistical analysis

Due to the repeated measures study design, appropriate statistical tests were chosen to analyze paired data. The association of host polymorphisms and *P. falciparum *presence in no surveys, one survey and two surveys was assessed by ordinal regression with adjustments for age. Differences in frequency of positive slides were assessed using χ^2 ^tests for each study year. Linear mixed models were used to examine the variation of parasite density among host genotypes with adjustments for age. Linear mixed models were also used to investigate the effect of age, parasite density and genotype on antibody levels: the two values were treated as a repeated measure with a heterogeneous compound symmetry covariance structure. Since the antibody levels showed considerable heteroscedasticity, they were transformed before analysis, using the transformation log(antibody + 2.5). The *P. falciparum *counts were transformed by log(*P. falciparum *+ 1) to reduce skewness while leaving the zero values unchanged. The models examined the effect of *P. falciparum *(logarithm of density), age (continuous), and either α^+^-thalassaemia genotype, CR1 genotype, ABO blood group (four categories [A, B, AB, O] and two categories [O and non-O]), SAO genotype or G6PD phenotype (three categories [normal, intermediate, deficient] and two categories [normal, abnormal]). Interactions between host erythrocyte polymorphisms were also examined but some subgroups obtained were too small for reliable analysis. An interaction between sex and G6PD deficiency was also examined as G6PD deficiency is an X-linked disorder [77]. CR1 expression has been shown to be associated with α^+^-thalassaemia genotype [5], therefore adjustments were made for the respective polymorphism in ordinal regression and mixed linear analysis. Addition of each polymorphism in each analysis did not alter the final result so no adjustments were made in the final models. The models were developed using R 1.8.1 [78] and SPSS (version 12.0) and variables with p-values < .05 were considered significant.

Informed consent was obtained from subjects and their parents or guardians. The project received ethical approval from the PNG Medical Research Advisory Committee and Institutional Review Board of the New York University School of Medicine.

## Results

### Characteristics of study cohort

1,100 blood samples and thick smears were collected from 555 children aged 1–17 years (mean age 9.68 ± 0.18) in the Amele region, Madang Province, PNG at two surveys in November-December of 1999 and 2000. Thirty-eight percent of samples were slide-positive for *P. falciparum *in both years of the study and 24.7% were positive for *Plasmodium vivax*. Only 57 samples (5.2%) were positive for both *P. falciparum *and *P. vivax*. Geometric mean parasite density of *P. falciparum *was 680 parasites/μl (95% CI 567–814) and parasite density was negatively correlated with age (*β *= -0.08, standard error [SE] 0.02, *P *= .002).

Table 1 details the frequency of polymorphisms investigated in this study. High frequencies of α^+^-thalassaemia were found in this population (92.9%) with a predominance of the 4.2 deletion allele (frequency = 0.71). The CR1 L allele was also predominant with a prevalence of 94.6%, whereas the host erythrocyte polymorphisms SAO and G6PD were relatively uncommon in this population (< 10%).

**Table 1:** Host polymorphism and parasitological data for 555 children from the Amele region, Papua New Guinea

		*P. falciparum *Prevalence^a^			
			
Host Polymorphism		Negative	Positive	Positive	
	Frequency	Both surveys	One survey	Both surveys	*P^b^*
	n (%)	n (%)	n (%)	n(%)	
α+-Thalassaemia					
(αα/αα)	39 (7.1)	15 (38.5)	19 (48.7)	5 (12.8)	.
(-α/αα)	205 (36.9)	75 (37.1)	94 (46.5)	33 (16.3)	0.72
(-α/-α)	311(56.0)	125 (41.7)	120 (40.0)	55 (18.3)	0.99
CR1^c^					
HH	29 (5.4)	14 (48.3)	12 (41.4)	3 (10.3)	.
HL	188 (35.5)	70 (38.0)	78 (42.4)	36 (19.6)	0.07
LL	313 (59.1)	121 (39.9)	133 (43.9)	49 (16.2)	0.11
ABO^c^					
O	180 (32.6)	69 (39.4)	66 (37.7)	40 (22.9)	.
non-O	372 (67.4)	145 (39.9)	165 (45.5)	53 (14.6)	0.09
A	178 (32.2)	62 (35.6)	80 (46.0)	32 (18.4)	0.83
B	138 (25.0)	62 (46.6)	58 (43.6)	13 (9.8)	0.005
AB	56 (10.1)	21 (37.5)	27 (48.2)	8 (14.3)	0.27
SAO^c^					
Normal	506 (91.2)	200 (40.4)	211 (42.6)	84 (17.0)	.
SAO	49 (8.8)	15 (32.6)	22 (47.8)	9 (19.6)	0.29
G6PD^c^					
Normal	477 (89.7)	182 (39.1)	199 (42.7)	85 (18.2)	.
Intermediate	20 (3.8)	8 (44.4)	6 (33.3)	4 (22.2)	0.76
Deficient	35 (6.6)	16 (45.7)	16 (45.7)	3 (8.6)	0.36

^*a *^*Missing *data: 1999, n = 1; 2000, n = 21.

^*b *^Differences in frequency of positive slides were assessed using ordinal regression with adjustments for age.

^c ^Abbreviations: CR1, Complement receptor 1; SAO, Southeast Asian Ovalocytosis; G6PD, Glucose-6-phosphate dehydrogenase deficiency. With the exception of SAO, these variables contain missing values.

### Host polymorphisms and *P. falciparum *prevalence and density

Table 1 also details whether a child was *P. falciparum *positive for one survey, two surveys or no surveys, according to host polymorphism. There was no association of α^+^-thalassaemia (*P *> .7), SAO (*P *= .29) or G6PD deficiency (*P *> .3) with *P. falciparum *prevalence either at both surveys, after adjustments for age. There was also no significant association of α^+^-thalassaemia (*P *= .78), SAO (*P *= .66) or G6PD deficiency (*P *= .64) with mean parasite density, after adjustments for age. Children who were blood group B were less likely to be positive at both surveys compared to those who were blood group O (*P *= .005). When *P. falciparum *prevalence was analysed separately for each year, blood group B had a significantly lower prevalence of *P. falciparum *(27.8%) compared to O (43.3%) in 2000 only (*P *= .02). There was no significant difference in mean parasite densities between ABO blood groups (*P *≤ .4) as assessed by a mixed linear model, with adjustments for age. Children with the CR1 HL genotype were more likely to be *P. falciparum *positive for both surveys compared to HH individuals but this was of borderline significance (*P *= .07). There was also weak evidence of CR1 HH genotype having lower geometric mean parasite densities (322 *P. falciparum*/μl, 95%CI 129–807) compared to those who were HL (786 *P. falciparum*/μl, 95% CI 584–1058, *P *= .07) or LL genotype (688 *P. falciparum*/μl, 95% CI 540–876, *P *= .12).

### Host polymorphisms and age-specific seroconversion to VSA

**Figure 1:**
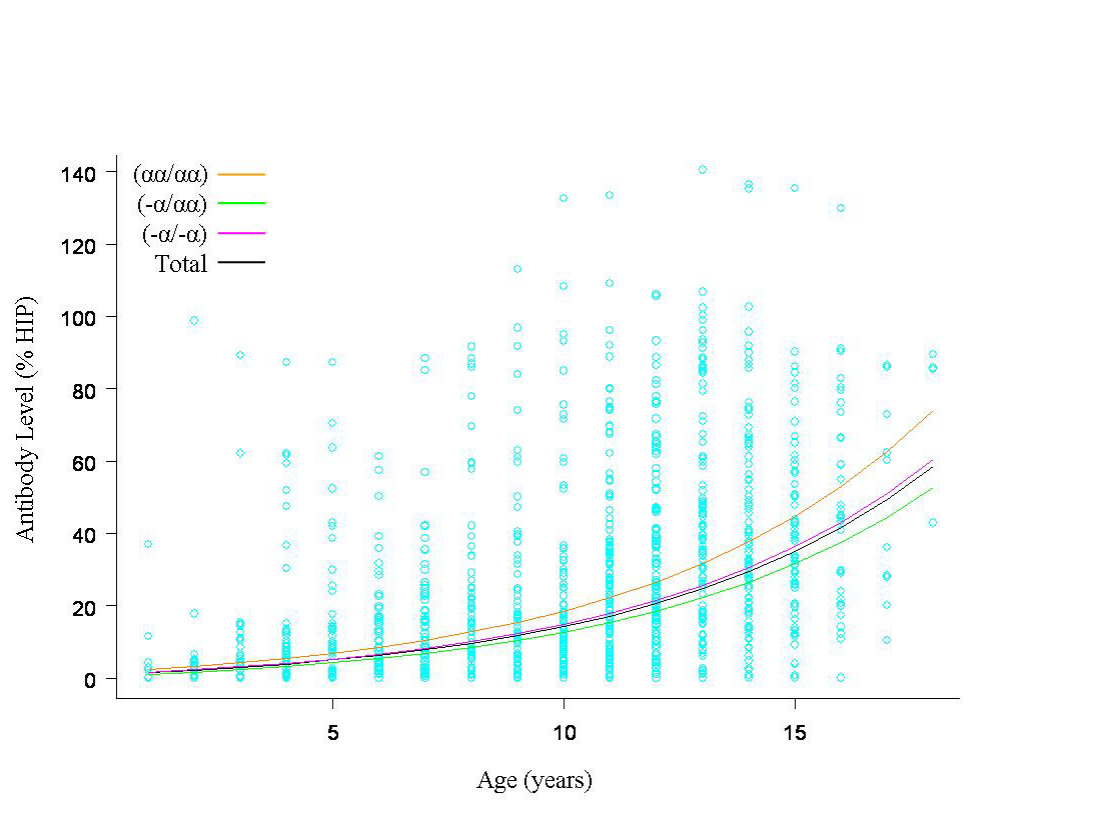
**Age-specific seroconversion to *P. falciparum*-infected erythrocyte variant surface antigens according to α^+^-thalassaemia genotype**. Antibody levels are expressed as the percentage relative response to a hyper-immune plasma control (HIP). Data represent the predicted antibody levels from a mixed linear model including age, *P. falciparum *parasitaemia (with parasitaemia set to 0) and α^+^-thalassaemia genotype. There was statistical significance between children of normal genotype and children heterozygous for α^+^-thalassaemia only (*P *= 0.02).

A multivariate linear mixed model was used to examine the effect of age and parasitaemia on antibody levels in samples from the cohort. Both age (*β *= 0.16, SE 0.009, *P *< .001) and parasitaemia (*β *= 0.03, SE 0.007, *P *< .001) were found to positively influence antibody levels. This model was then applied to each of the five host erythrocyte polymorphisms investigated in this study. There was no significant difference in the age-dependent acquisition of VSA-specific antibodies with respect to ABO blood groups (*P *≥ .39), SAO (*P *= .39), or CR1 genotype (*P *= .99). There was also no significant difference in age-dependent seroconversion in children with G6PD deficiency (*P *> .2), nor was there evidence of an interaction between G6PD deficiency and sex (*P *= .16). There was an effect of α^+^-thalassaemia on age-dependent anti-VSA antibody acquisition (Figure 1). Children who were heterozygous for α^+^-thalassaemia had a slower rate of antibody acquisition compared to children of normal genotype (*P *= .02). There was no significant difference in rate of antibody acquisition between children of normal genotype and those that were homozygous for α^+^-thalassaemia (*P *= .14).

## Discussion

This study investigated age-specific antibody seroconversion in Amele villages on the north coast of PNG, where malaria transmission is intense [70]. The sample size of the study was sufficiently large to allow us to look for interactions among the most common polymorphisms. Age was positively correlated with seroconversion to VSA, reflecting the association of age with exposure to malaria [66]. Parasitaemia was also positively correlated with VSA antibody levels. This result is similar to what has previously been shown in asymptomatic African populations to heterologous VSA [79]. It is therefore essential to make appropriate adjustments for age and parasitaemia when examining the association of host erythrocyte polymorphisms with age-specific antibody seroconversion.

Papua New Guinean children who are homozygous, but not heterozygous, for α^+^-thalassaemia are protected against severe malaria [1]. These children have significantly smaller erythrocytes compared to children of normal genotype, whereas erythrocyte parameters in heterozygotes are indistinguishable from normal individuals [80]. If there is a reduced physical interaction (i.e reduced invasion/growth, rosetting) or enhanced immune-mediated response between microcytic erythrocytes and *P. falciparum*, then a difference in parasite prevalence/density and seroconversion would be expected between homozygous children and those of normal genotype. This study showed there was no significant difference in age-specific seroconversion among these genotypes. Furthermore, there was no significant difference in *P. falciparum *density between α^+^-thalassaemia genotypes which is in concordance with other studies in children with asymptomatic parasitaemia [4,11,60-62]. There was some evidence of children of normal genotype having increased exposure compared to children who were heterozygous, but not homozygous, for α^+^-thalassaemia. This was surprising given that erythrocyte indices for heterozygotes are similar to children of normal genotype [80]. There is strong evidence that α^+^-thalassaemia confers particular protection against severe malarial anaemia. This must be independent of parasite density which has shown to be similar among α^+^-thalassaemia genotypes in children with acute malaria [1-4].

CR1 deficiency and blood group O are thought to protect against malaria by decreased rosetting. A positive correlation has been shown between rosetting and *P. falciparum *parasitaemia and it has been proposed that rosetting may enhance parasite survival by facilitating invasion and promoting immune evasion [81]. Polymorphisms associated with increased rosetting such as the CR1 H allele and AB blood antigens should lead to increased parasitaemia by this proposed mechanism. Contrary to this expectation, this study showed weak evidence of children with high levels of CR1 expression (HH) having lower *P. falciparum *prevalence and densities compared to children with lower CR1 expression (HL or LL genotype). This result could have occurred by chance given that only 15 children with HH were *P. falciparum *positive during the study. There was also evidence of blood group B having lower *P. falciparum *prevalence in the 2000 survey, but there was no significant difference in parasite densities among ABO blood groups. Neither ABO blood group and CR1 genotype had no effect on age-dependent seroconversion to anti-VSA antibodies, indicating that differences in parasitological data in these groups must have been due to chance. This study would support the hypothesis that ABO blood group and CR1 deficiency associated with the L allele protect against malaria by reduced rosetting and associated pathology rather than parasite density.

There was no significant difference in geometric mean parasite densities or rates of seroconversion to *P. falciparum *VSA with respect to G6PD and SAO genotypes. These data conflict with several studies that have proposed that innate resistance to malaria involved the inability for the malaria parasite to grow in and/or invade G6PD deficient and SAO erythrocytes [23]. There was also no evidence of an interaction between G6PD deficiency and sex with respect to anti-VSA seroconversion. A recent case-control study demonstrated that males, but not females, with G6PD deficiency were protected against severe malaria [12], but there was no significant difference in parasite density. Results presented here concur with this latter observation.

## Conclusion

Data presented here highlight the importance of measuring malaria exposure at two time points to investigate the interactions of host polymorphisms with the malaria parasite. Results can occur by chance, and previous studies have reported data from a single time point. No polymorphism showed both a reduction in parasite prevalence/density and age-specific seroconversion to anti-VSA antibodies. We interpret these findings to indicate that the mechanisms of protection by these polymorphisms are not fully explained by either structural aspects of the erythrocyte preventing infection or by enhanced immune responses to VSA. Our observations from PNG differ significantly from those we and others have seen for African children with HbAS and/or Hb C carriers. HbAS individuals have lower parasite prevalence/densities and delayed time to reinfection, as well as increased prevalence of anti-VSA antibodies, compared to those of normal genotype [18,24,36-39,44,82,83]. Whereas HbC carriers exhibit no differences in parasite density, yet higher immune responses to VSA [83]. The enhanced immune reactivity in Hb S and HbC carriers would suggest that protection against malaria is partially mediated by acquired immunity. Conversely, the polymorphisms investigated in PNG may confer protection against the clinical pathologies of *P. falciparum *infection by a mechanism that does not influence parasite density or immunity to VSA.

## Authors' contributions

FJIF performed data analysis, interpreted data and prepared the final manuscript with KPD. PM helped to draft the manuscript and undertook laboratory work (FACS assay and PCR-based host genotyping except CR1). LP and CMW helped undertake laboratory work and interpret data. HI and LT undertook laboratory and field work. RR participated in the design of analysis. CSM, AJFL and KPD conceived the study, participated in its design, and obtained funding for the project. All authors discussed the results and approved the final manuscript.
